# MicroRNA Expression Profiles Before and After Neoadjuvant Chemotherapy in Breast Cancer: Correlations with Molecular Subtypes, Pathological Response, and Clinical Timing—A Pilot Translational Study

**DOI:** 10.3390/ijms27177799

**Published:** 2026-08-31

**Authors:** Isabela Anda Komporaly, Adelina Silvana Gheorghe, Elena Adriana Iovănescu, Bogdan Georgescu, Dana Lucia Stănculeanu

**Affiliations:** 1 Department of Oncology, “Carol Davila” University of Medicine and Pharmacy, 020021 Bucharest, Romania; isabelakomporaly@gmail.com (I.A.K.); dumitrescu.elena.adriana@gmail.com (E.A.I.); dr_bogdan.georgescu@icloud.com (B.G.); dlstanculeanu@gmail.com (D.L.S.); 2 Memorial Băneasa Hospital, 013812 Bucharest, Romania; 3 Department of Medical Oncology I, “Prof. Dr. Alexandru Trestioreanu” Institute of Oncology, 022328 Bucharest, Romania; 4 Neolife Hospital, 077190 Bucharest, Romania

**Keywords:** breast cancer, microRNA, neoadjuvant chemotherapy, small-RNA sequencing, residual cancer burden, pathological complete response, molecular subtypes, biomarkers, translational oncology

## Abstract

Neoadjuvant chemotherapy (NAC) is the standard of care for locally advanced breast cancer, yet the molecular predictors of pathological response remain incompletely defined, particularly regarding microRNA (miRNA) dynamics. We investigated paired pre- and post-NAC miRNA expression profiles in relation to molecular subtype, residual cancer burden (RCB), and clinical timing parameters. Seven patients with invasive breast cancer (Luminal A *n* = 3, Luminal B *n* = 1, TNBC *n* = 2, HER2+ *n* = 1) who received NAC (AC-T or TCHP) were included in this pilot study. Small-RNA sequencing (NovaSeq X Plus, CeGaT GmbH, project S17293) was performed on 14 FFPE specimens (7 pre-NAC core needle biopsies, 7 post-NAC surgical specimens). Differential expression analysis used the paired Wilcoxon signed-rank test with Benjamini–Hochberg correction. Spearman correlations assessed associations between miRNA expression, RCB score, and clinical timing intervals. Candidate miRNAs were subsequently annotated using experimentally validated miRNA–target interactions. After filtering (≥ three counts in ≥ three samples), 759 miRNAs were analysed. No miRNA reached strict significance (*p*adj < 0.05, |log_2_FC| > 1.0) after multiple testing correction, consistent with the limited statistical power (*n* = 7). Under exploratory criteria (*p* < 0.10, |log_2_FC| > 0.5), 156 candidate miRNAs were identified: 70 upregulated and 86 downregulated post-NAC. Leading candidates included hsa-miR-139-3p (+2.60), hsa-miR-139-5p (+2.36), and hsa-miR-1323 (+1.55) as upregulated, and hsa-miR-429 (−2.53), hsa-miR-141-3p (−1.79), and hsa-miR-1277-5p (−1.25) as downregulated post-NAC. The single patient achieving the lowest residual disease burden (P3, Luminal B, RCB-I, score 1.32) displayed a distinct pre-treatment miRNA profile, separating from all other pre-NAC specimens on principal component analysis and characterised by higher baseline hsa-miR-139-3p/-5p and lower baseline hsa-miR-429 and hsa-miR-141-3p expression, suggesting that baseline miRNA expression patterns may contribute to differential chemotherapy response. RCB score showed a non-significant positive trend with post-NAC Ki-67 (ρ = +0.71, *p* = 0.07). This pilot study identifies NAC-modulated candidate miRNAs in breast cancer and establishes a paired FFPE-based small-RNA-sequencing workflow applicable in routine clinical settings. The distinct pre-treatment profile of the single best responder generates the testable hypothesis that baseline expression of tumour suppressor miRNAs of the miR-139 family, together with low miR-200-family expression, may track chemosensitivity. As no candidate reached statistical significance after multiple testing correction and none has been validated in an independent cohort or by an orthogonal method, all findings are exploratory and hypothesis-generating. The results support larger prospective validation studies examining miRNA signatures as predictive biomarkers of NAC response across breast cancer molecular subtypes.

## 1. Introduction

Breast cancer remains the most frequently diagnosed malignancy and the leading cause of cancer mortality among women worldwide, with an estimated 2.3 million new cases diagnosed in 2022 [1,2]. Neoadjuvant chemotherapy (NAC) has evolved from a strategy to render inoperable tumours resectable into a standard-of-care approach in locally advanced and high-risk early breast cancer, offering the unique advantage of providing an in vivo assessment of treatment sensitivity through evaluation of the pathological response in the surgical specimen.

Pathological complete response (pCR), defined as the absence of residual invasive carcinoma in both the breast and the axillary lymph nodes (ypT0/is ypN0), has been established as a surrogate endpoint for long-term survival, particularly in HER2-positive and triple-negative breast cancer (TNBC) subtypes [3,4]. The Residual Cancer Burden (RCB) system, developed at the MD Anderson Cancer Center, provides a more granular quantification of residual disease and demonstrates superior prognostic discrimination compared with the binary pCR assessment across all molecular subtypes [5].

Despite improvements in NAC regimens—including dual HER2 blockade (trastuzumab + pertuzumab) achieving pCR rates of 45–66% in HER2-positive disease, and pembrolizumab-containing regimens reaching 64.8% pCR in TNBC (KEYNOTE-522)—a substantial proportion of patients present with residual disease after NAC, which is associated with a significantly worse prognosis [6,7]. Identifying molecular biomarkers that predict NAC response before treatment initiation, or that dynamically monitor response, would substantially improve patient management by enabling treatment escalation or de-escalation strategies.

MicroRNAs (miRNAs)—small, 18–25-nucleotide non-coding RNA molecules that regulate gene expression mainly through post-transcriptional repression of target mRNAs—have emerged as compelling biomarker candidates in breast cancer oncology [8,9]. Their stability in formalin-fixed, paraffin-embedded (FFPE) tissue, their detectability in serum and plasma, and their involvement in critical oncogenic pathways (PI3K/AKT, MAPK, EMT, apoptosis) make them particularly attractive for translational research [10]. Multiple studies have implicated specific miRNAs—including miR-21, miR-155, the miR-200 family, and miR-139-3p/5p—in chemoresistance mechanisms and treatment response prediction, although clinical validation remains limited by small cohort sizes and methodological heterogeneity [11,12].

The present pilot study addresses this gap by characterising paired pre- and post-NAC miRNA expression profiles using small-RNA sequencing on FFPE specimens from patients treated with contemporary NAC regimens, and by correlating miRNA expression patterns with molecular subtype, RCB class, and clinical timing parameters. To our knowledge, this is among the few paired small-RNA-sequencing studies on FFPE specimens performed in a real-world Romanian oncology cohort, providing foundational data for the design of adequately powered prospective validation studies.

## 2. Results

### 2.1. Patient Characteristics

**Figure 1:**
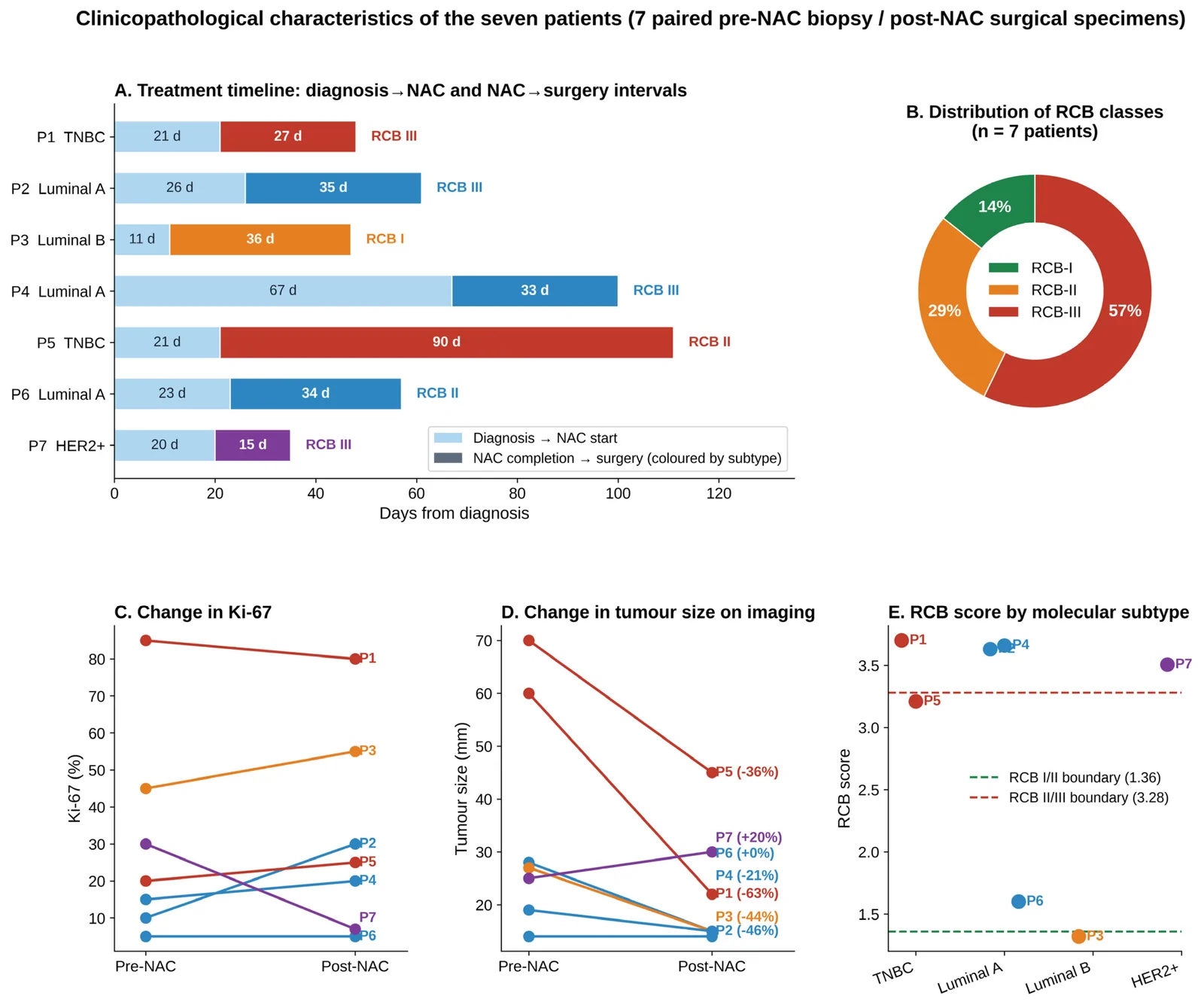
Clinical overview of the seven patients included in the study. (**A**) Treatment timeline showing the intervals from diagnosis to NAC initiation and from NAC completion to surgery, per patient. (**B**) Distribution of RCB classes. (**C**) Pre- vs. post-NAC Ki-67 changes per patient and molecular subtype. (**D**) Imaging changes in tumour size. (**E**) RCB scores by molecular subtype. Colour coding: red = TNBC, blue = Luminal A, orange = Luminal B, purple = HER2+. RCB thresholds: green dashed line = RCB I/II boundary (1.36); red dashed line = RCB II/III boundary (3.28).

Seven patients with invasive breast carcinoma of no special type (NST) who received NAC between 2021 and 2025 were included (Table 1). The cohort comprised three Luminal A patients, one Luminal B patient, two TNBC patients, and one HER2-positive patient. All tumours were grade G2–G3, with Ki-67 ranging from 5 to 85%. Six patients received AC-T (anthracycline–taxane) and one received TCHP. No patient achieved a pathological complete response (pCR rate 0/7); the distribution of RCB classes was RCB-I (*n* = 1, 14.3%), RCB-II (*n* = 2, 28.6%), and RCB-III (*n* = 4, 57.1%). The median interval from diagnosis to NAC initiation was 21 days (range 11–67), and that from NAC completion to surgery was 34 days (range 15–90). The clinical timeline is illustrated in Figure 1.

### 2.2. RNA Quality Control and Sequencing Metrics

RNA quality from the FFPE specimens varied as expected. Across the 14 included specimens, RIN values ranged from 1.0 to 6.7, and DV200 values were above 20%, consistent with the expected quality of FFPE-derived RNA used for miRNA sequencing. One additional specimen (S17293Nr35), belonging to a patient outside the present cohort, had a DV200 of 2.3%, failed quality control, and was excluded before sequencing; it is not included in any of the values reported above.

Sequencing depth was uniform across the 14 samples: 10.106–10.110 million reads per sample, with a mean Q30 of 92.76%, indicating excellent sequencing quality. After miRge3.0 alignment, 517–656 unique miRNAs were detected per sample (median 612), with most reads mapping to mature miRNAs (mean mapping rate 62%).

Novel-miRNA predictions were generated in 6 of 14 samples (0–2 per sample, probability > 0.96). These are unvalidated algorithmic predictions and were not carried forward into any downstream analysis.

### 2.3. Principal Component Analysis

**Figure 2:**
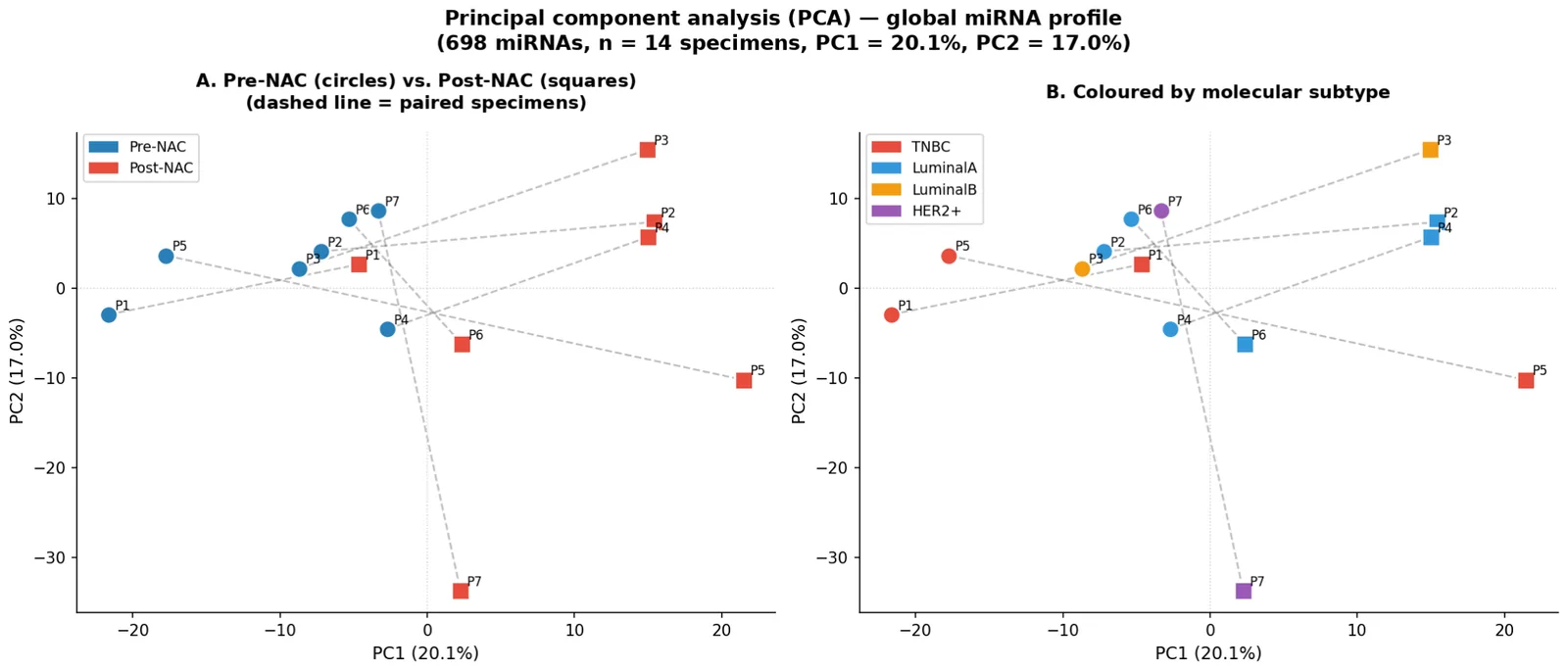
Principal component analysis (PCA) of log_2_(RPM+1)-transformed miRNA expression profiles for 14 samples (7 pre-NAC, 7 post-NAC). (**A**) Pre-NAC (circles) vs. post-NAC (squares) samples; dashed lines connect paired specimens. (**B**) The same PCA coloured by molecular subtype. The variance explained by PC1 and PC2 is indicated on the axes. Paired pre/post samples from the same patient tend to cluster closely, reflecting the dominant patient-specific transcriptomic background.

PCA of the log_2_(RPM+1)-transformed expression values for the 14 samples (7 pre-NAC, 7 post-NAC) is shown in Figure 2. PC1 explained the predominant inter-sample variance, with pre- and post-NAC samples from the same patient generally occupying nearby positions in principal component space, consistent with patient-specific transcriptomic backgrounds dominating the global variability of miRNA expression. Patient P3 (Luminal B, RCB-I—the lowest residual disease burden) demonstrated the most distinct pre-NAC miRNA profile among the pre-treatment specimens, with the largest PC1 displacement relative to the other pre-NAC samples. The HER2+ patient (P7, TCHP regimen) showed the most pronounced shift between pre- and post-NAC positions, potentially reflecting the distinctive on-target engagement of dual HER2 blockade.

### 2.4. Differential miRNA Expression: Pre- vs. Post-NAC

**Figure 3:**
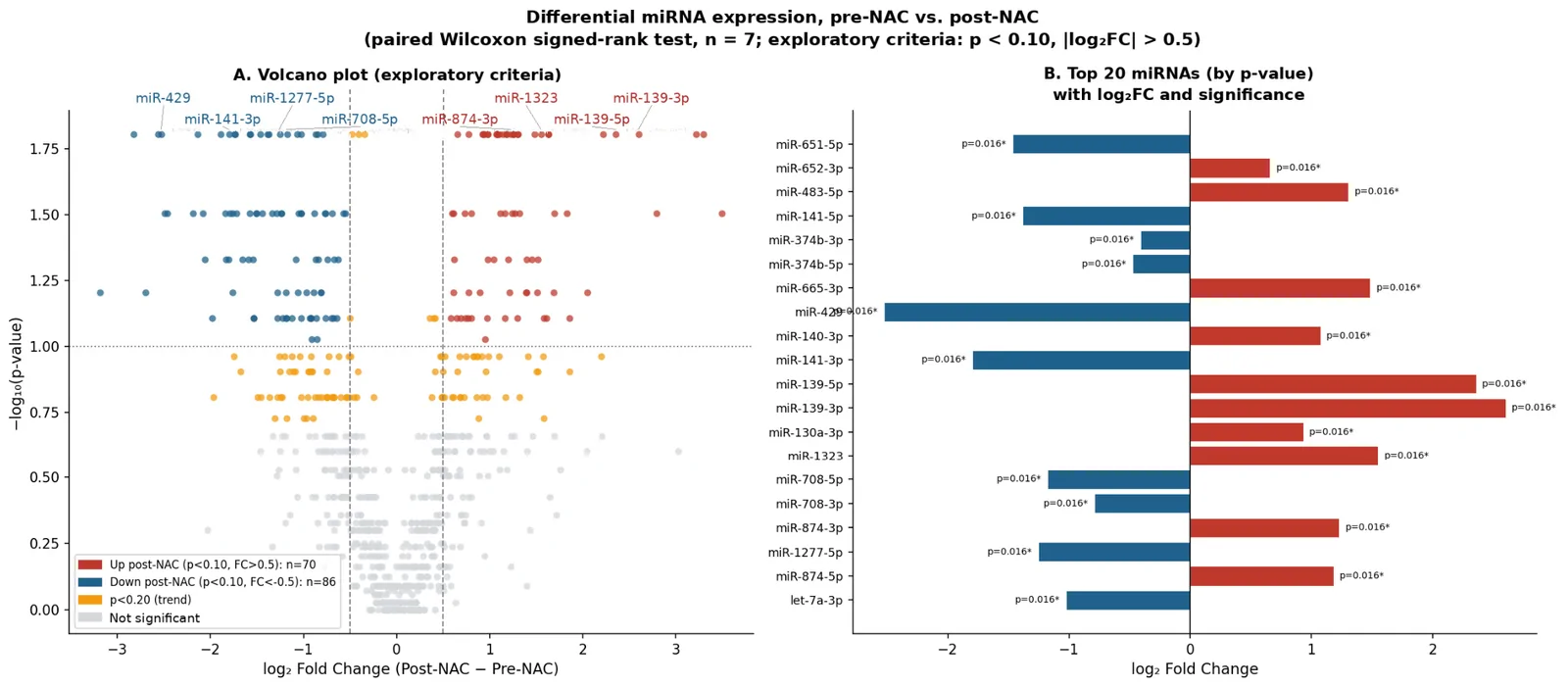
Differential miRNA expression between pre-NAC and post-NAC specimens. (**A**) Volcano plot: x-axis, log_2_ fold change (post-NAC − pre-NAC); y-axis, −log_10_ of the uncorrected *p*-value from the paired Wilcoxon signed-rank test. Note that at *n* = 7 pairs the Wilcoxon statistic is discrete and its *p*-value can take only a limited set of values (minimum 1/64 = 0.0156); points consequently align in horizontal bands, which is the expected geometry of this test at this sample size and not a plotting artefact. Vertical dashed lines: |log_2_FC| = 0.5 threshold. Horizontal dotted line: *p* = 0.10 threshold. Red points: upregulated post-NAC (*n* = 70); blue points: downregulated post-NAC (*n* = 86); orange: *p* < 0.20 trend; grey: non-significant. Only the ten leading candidates are labelled, for legibility. (**B**) The 20 miRNAs with the smallest uncorrected *p*-values, ranked by log_2_FC magnitude. No candidate reached *p*adj < 0.05 (Table S1). * *p* < 0.05.

After filtering for minimum expression (759 miRNAs), the paired Wilcoxon signed-rank analysis identified no miRNA meeting the strict significance criteria (*p*adj < 0.05, |log_2_FC| > 1.0) after BH correction for multiple testing—an expected finding given the inherent power limitation of *n* = seven pairs. Under the pre-specified exploratory criteria (uncorrected *p* < 0.10, |log_2_FC| > 0.5), 156 candidate miRNAs were identified: 70 upregulated (post vs. pre-NAC) and 86 downregulated. The leading candidates are displayed in Figure 3 (volcano plot and ranked bar chart).

The most strongly upregulated post-NAC miRNAs (by log_2_FC magnitude) included hsa-miR-139-3p (+2.60), hsa-miR-139-5p (+2.36), hsa-miR-1323 (+1.55), hsa-miR-874-3p (+1.23), and hsa-miR-874-5p (+1.18). The most strongly downregulated included hsa-miR-429 (−2.53), hsa-miR-141-3p (−1.79), hsa-miR-1277-5p (−1.25), hsa-miR-708-5p (−1.18), and hsa-let-7a-3p (−1.02). Notably, the miR-200-family member hsa-miR-429 and the EMT regulator hsa-miR-141-3p showed consistent post-NAC downregulation across all patients, whereas miR-139-3p/5p—previously reported as tumour suppressors targeting MMP-2, CDH11, and Notch signalling—were markedly upregulated, suggesting potential activation of antitumor mechanisms by NAC.

### 2.5. Heatmap and Per-Patient miRNA Expression Patterns

**Figure 4:**
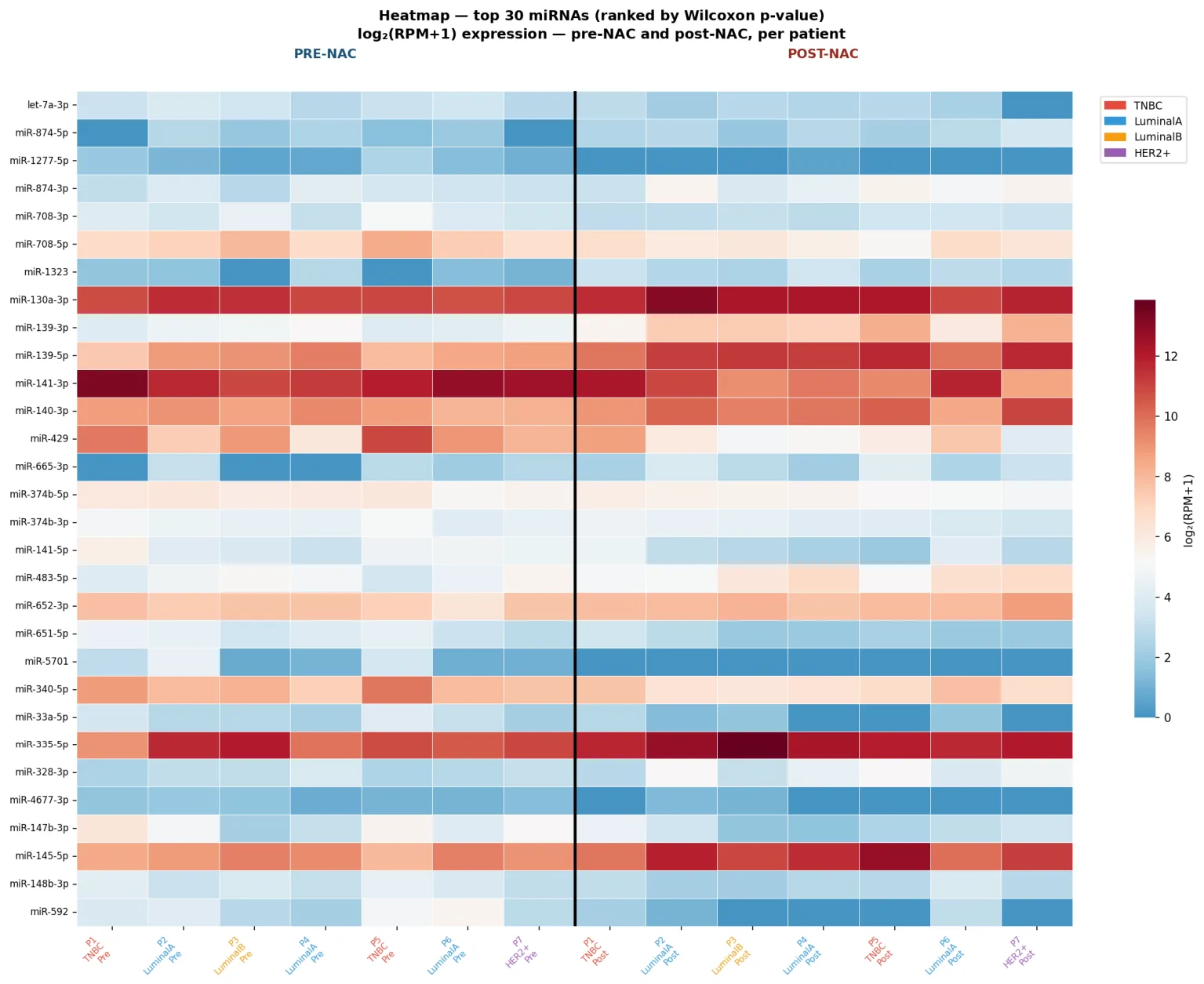
Heatmap of the top 30 miRNAs (ranked by Wilcoxon *p*-value) across all 14 samples. Colour scale: log_2_(RPM+1) expression values (red = high, blue = low). The vertical black line separates pre-NAC (**left**) and post-NAC (**right**) specimens. Column labels are colour-coded by molecular subtype. Rows represent individual miRNAs, sorted by significance rank.

The heatmap of the top 30 miRNAs (ranked by Wilcoxon *p*-value) across all 14 samples reveals distinct expression patterns that partially segregate by molecular subtype (Figure 4). The two TNBC patients (P1, P5) share relatively similar pre-NAC miRNA profiles, as do the three Luminal A patients (P2, P4, P6). The Luminal B patient (P3)—with the lowest RCB score—demonstrates a pre-NAC miRNA expression pattern characterised by higher miR-139-3p/5p expression and lower miR-429/141-3p expression compared with the other patients. This observation, although preliminary, is consistent with the hypothesis that pre-NAC expression of tumour suppressor miRNAs (the miR-139 family) may be associated with greater NAC sensitivity.

**Figure 5:**
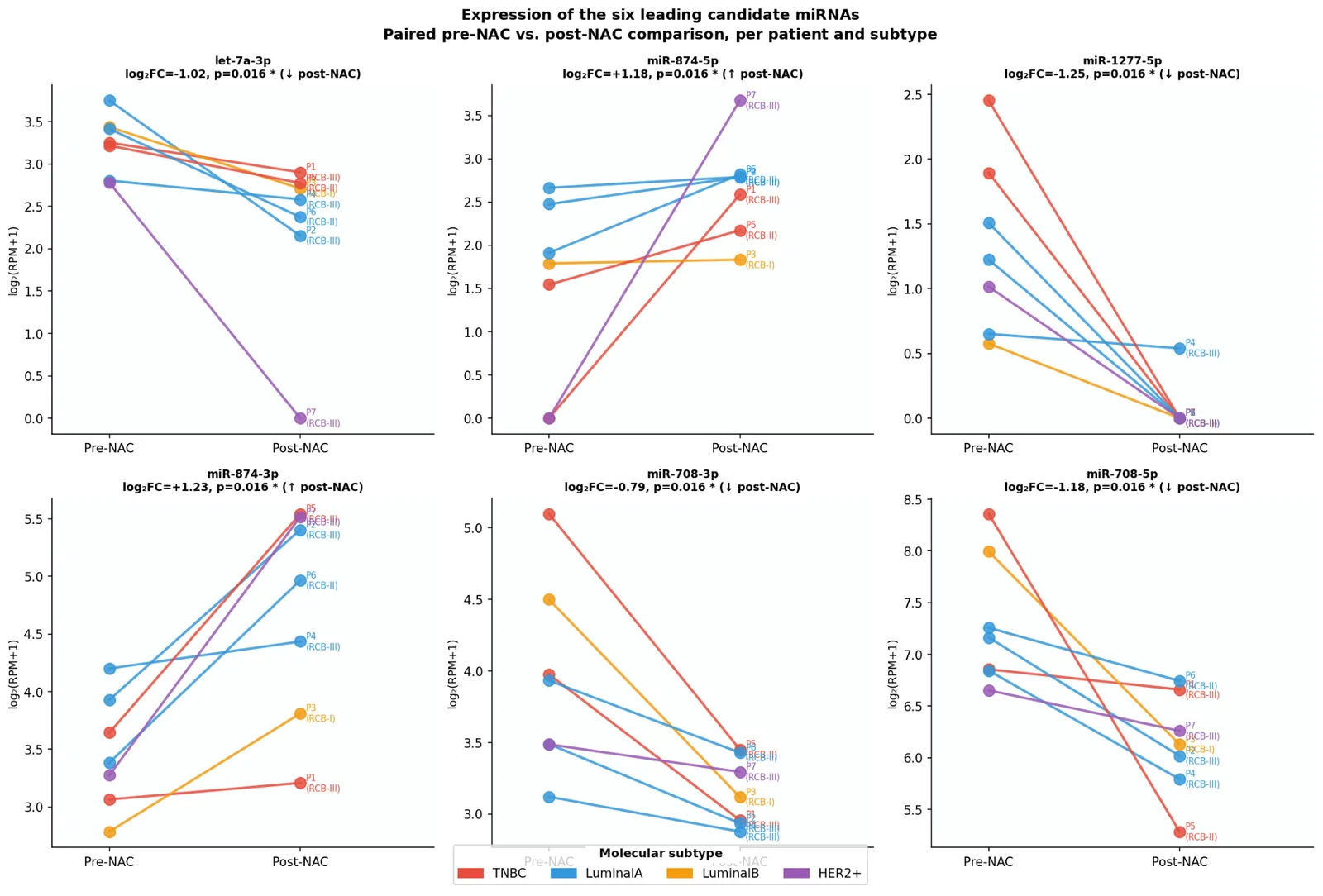
Paired expression trajectories for the top 6 candidate miRNAs across all 7 patients (pre-NAC to post-NAC). Each line connects the pre- and post-NAC log_2_(RPM+1) expression values for a patient, colour-coded by molecular subtype. Statistical annotation: * *p* < 0.05 (Wilcoxon signed-rank, uncorrected). The direction of change (↑/↓ post-NAC) and log_2_ fold change are indicated in the panel titles.

Paired expression trajectories for the top six differentially expressed miRNAs are shown in Figure 5. A notable observation is that the directionality of expression changes (up vs. down post-NAC) is generally consistent across patients for individual miRNAs, supporting the biological plausibility of these candidates despite the absence of statistical significance after multiple testing correction.

### 2.6. Correlations with RCB Score and Clinical Timing

**Figure 6:**
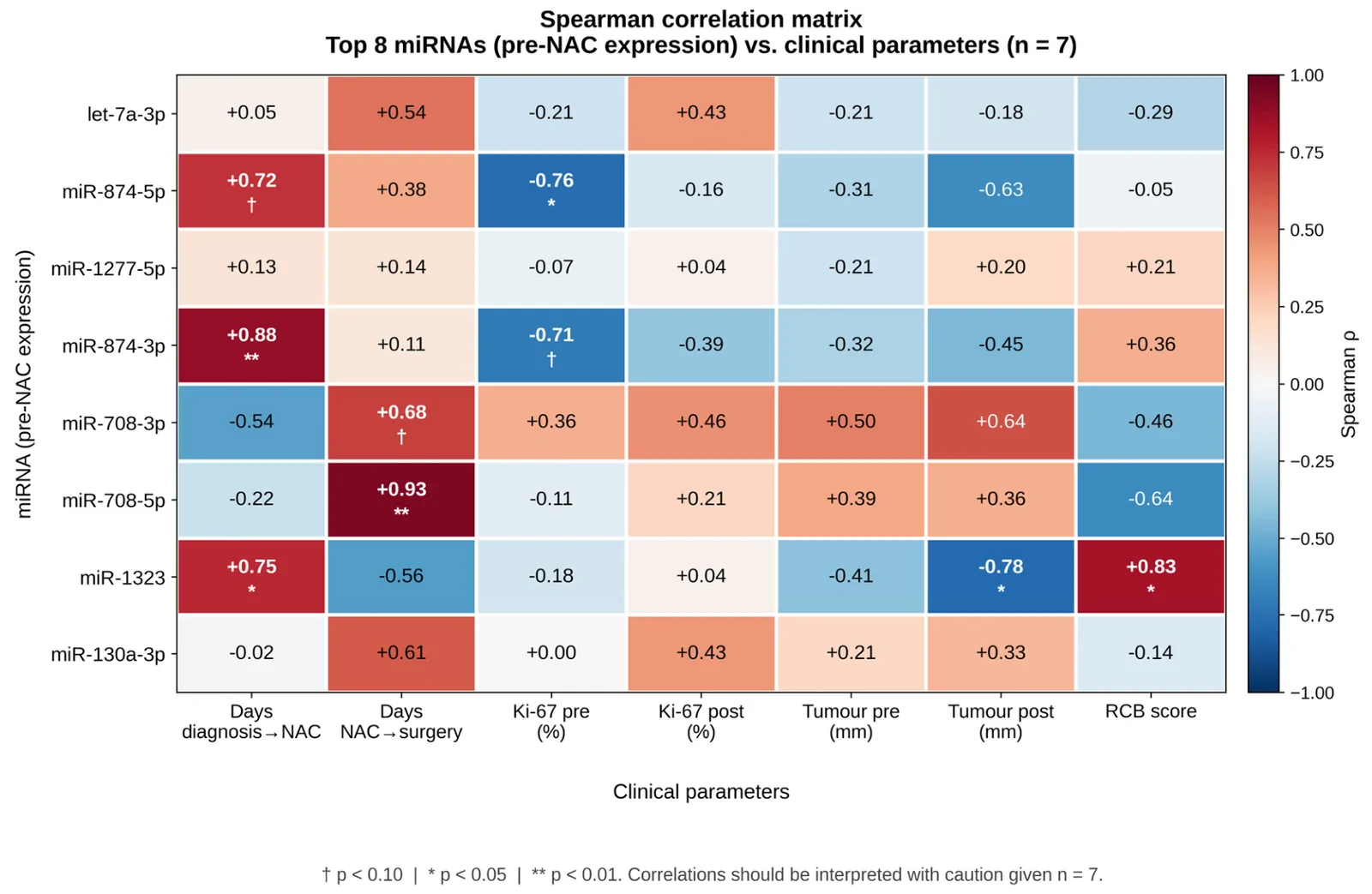
Spearman correlation matrix between pre-NAC miRNA expression levels for the top 8 candidates and clinical variables (*n* = 7 patients). Colour scale: red = positive correlation, blue = negative correlation (ρ range −1 to +1). The numerical values in each cell show the ρ coefficient and significance annotation († *p* < 0.10, * *p* < 0.05, ** *p* < 0.01). Statistical note: correlations should be interpreted with caution given *n* = 7.

**Figure 7:**
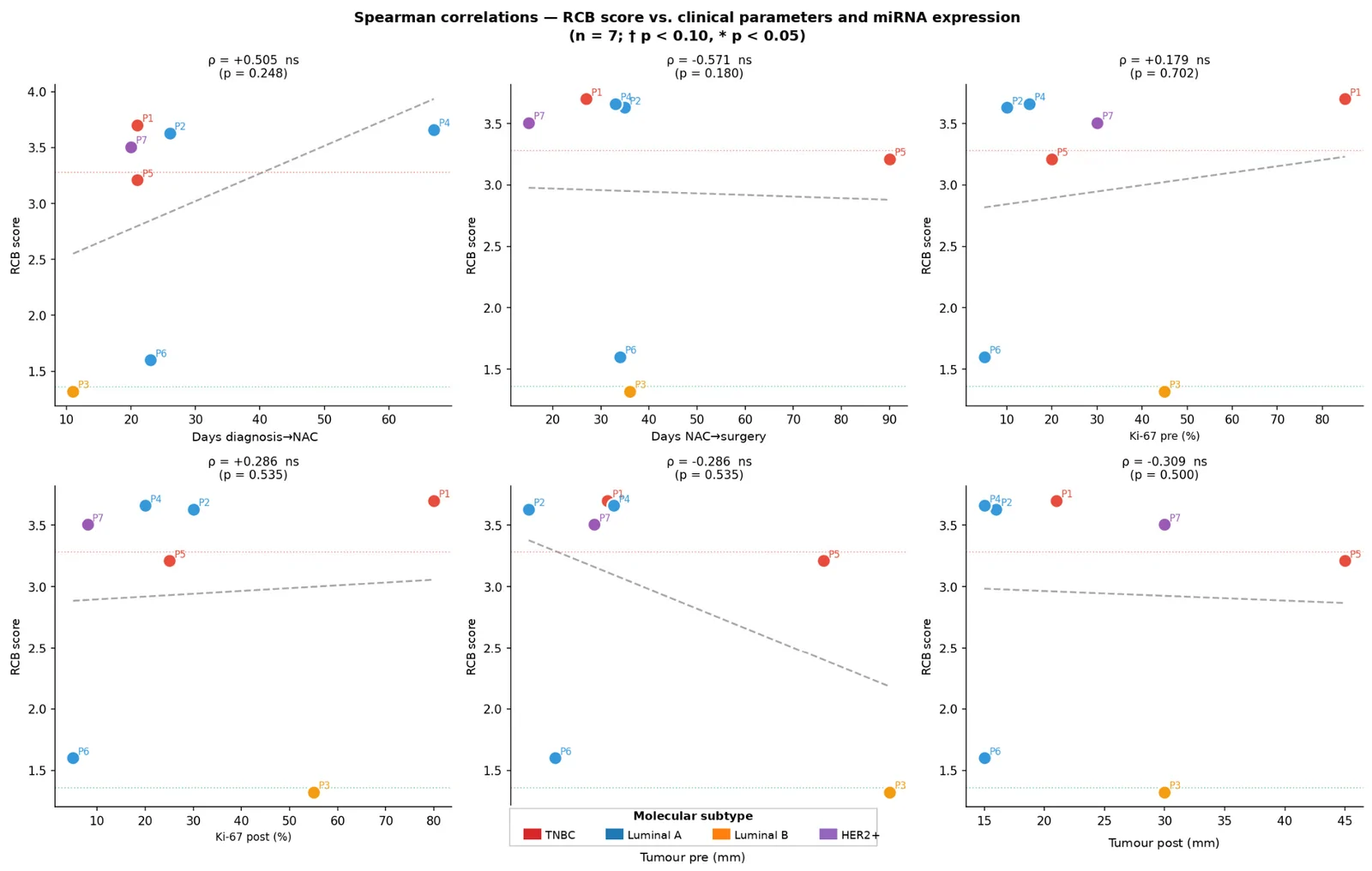
Spearman correlation scatter plots between RCB score and six clinical parameters (*n* = 7). Each point represents a patient, colour-coded by molecular subtype. Dashed regression lines indicate the direction of association. Dotted horizontal lines indicate RCB-class boundaries (green: I/II at 1.36; red: II/III at 3.28). The ρ and *p* values are reported in the panel titles. † *p* < 0.10, * *p* < 0.05.

Spearman correlations between pre-NAC miRNA expression (top eight candidates), RCB score, and clinical variables are summarised in Figure 6. Among the clinical variables, post-NAC Ki-67 showed the strongest (non-significant) positive correlation with RCB score (ρ = +0.71, *p* = 0.071), suggesting—as expected—that the proliferative activity of the residual post-NAC tumour tracks the extent of residual disease. Pre-NAC Ki-67 showed a moderate positive trend with RCB (ρ = +0.54, *p* = 0.21), whereas the clinical timing parameters (days from diagnosis to NAC initiation, days from NAC completion to surgery) showed no significant correlations with RCB score in this small cohort (Figure 7).

Notably, the interval from NAC completion to surgery (days NAC → surgery) showed a positive trend with RCB score (ρ = +0.50, *p* = 0.25), driven mainly by patient P5 (TNBC, 90 days NAC → surgery, RCB-II, score 3.21)—although the small sample size precludes any definitive conclusions. The prolonged preoperative interval in P5 resulted from logistical factors (patient scheduling and recovery from neutropenia) rather than disease progression, as confirmed by imaging.

### 2.7. Functional Annotation of the Candidate Signature

**Figure 8:**
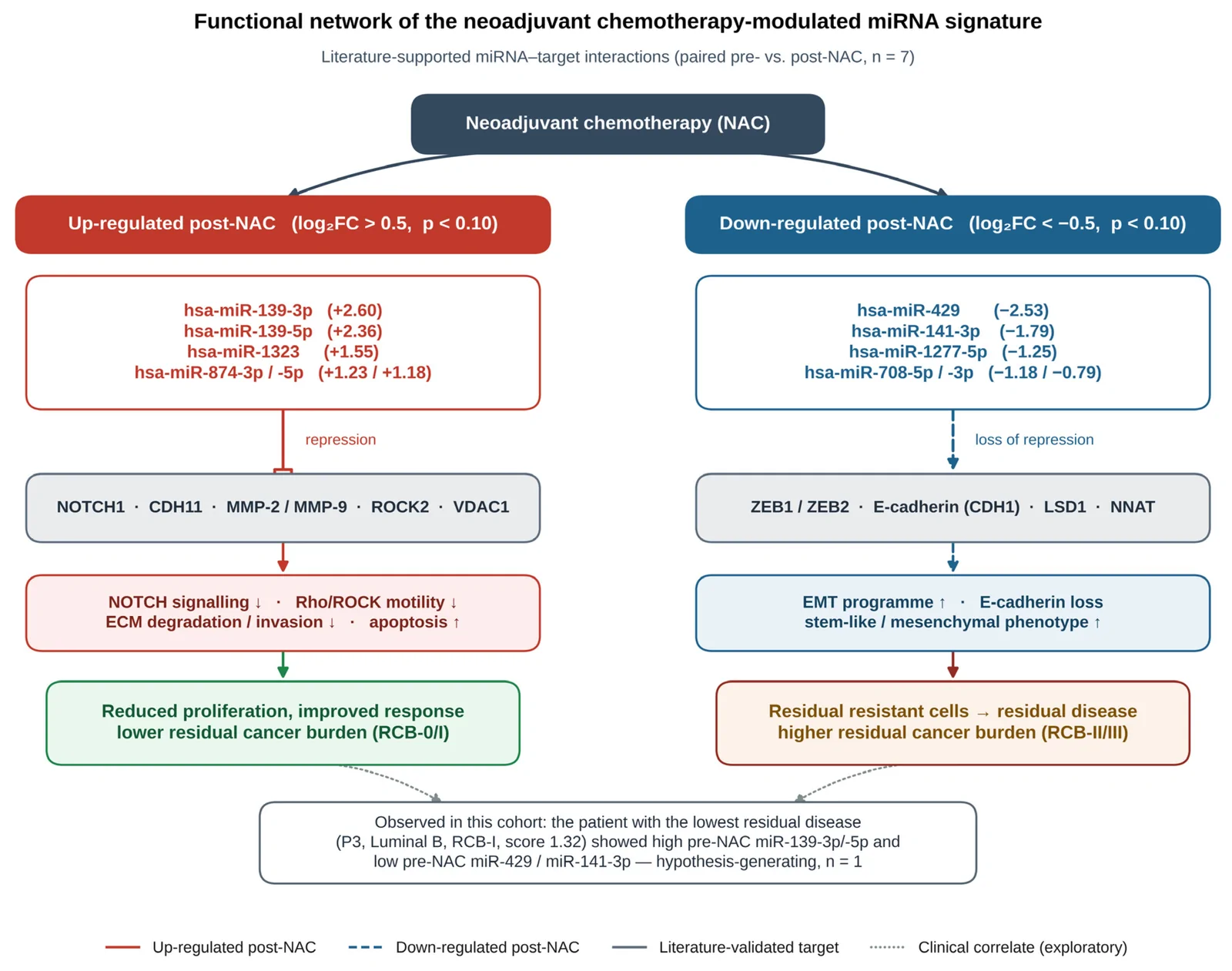
Proposed biological pathways associated with the candidate miRNA signature identified after neoadjuvant chemotherapy. (**Left**): miRNAs upregulated in post-NAC specimens (log_2_FC > 0.5, uncorrected *p* < 0.10) and their experimentally validated targets, with the predicted consequences for proliferation and treatment response. (**Right**): miRNAs downregulated post-NAC and the targets that are consequently de-repressed, with the predicted consequences for EMT and residual disease. Bar-headed connectors indicate repression; arrowheads indicate loss of repression. Bottom: the corresponding clinical observation in this cohort (*n* = 1, hypothesis-generating). Only interactions supported by strong experimental evidence in miRTarBase 9.0 and the primary literature are shown [14,15,16,17,18,19,20]; hsa-miR-1323 is included for completeness of the signature although validated targets in breast cancer are currently lacking. This diagram summarises published target biology and is not the output of a statistical enrichment analysis; no candidate miRNA in this study reached significance after multiple testing correction.

The leading candidates converge on two functionally coherent programmes (Figure 8). The miRNAs upregulated after NAC (miR-139-3p/-5p, miR-874-3p/-5p) are established repressors of NOTCH1, CDH11, MMP-2/MMP-9, ROCK2, and VDAC1, that is, of pathways governing NOTCH signalling, Rho/ROCK-dependent motility, extracellular matrix degradation, and cell invasion. The miRNAs downregulated after NAC (miR-429, miR-141-3p, miR-1277-5p, miR-708-5p/-3p) include members and functional relatives of the miR-200 family, whose loss de-represses ZEB1/ZEB2 and permits E-cadherin downregulation and acquisition of a mesenchymal, stem-like phenotype. The two programmes are therefore directionally opposite with respect to predicted residual tumour behaviour, and the residual tumours in this cohort (six of seven with RCB-II/III) displayed the downregulated miR-200 pattern uniformly across all patients.

## 3. Discussion

### 3.1. Principal Findings and Study Framing

This study was intentionally designed as a pilot feasibility study aimed at evaluating the applicability of paired FFPE small-RNA sequencing in routine clinical samples and at generating candidate biomarkers, rather than at establishing definitive predictive signatures. Read with that intent, it presents, to our knowledge, one of the relatively few paired pre/post-NAC small-RNA-sequencing analyses on FFPE specimens from a real-world Eastern European breast cancer cohort.

The key findings are: (1) successful small-RNA-sequencing profiling from FFPE biopsies with DV200 ≥ 20%; (2) identification of 156 exploratory candidate miRNAs differentially expressed post-NAC; (3) consistent upregulation of miR-139-3p/5p and downregulation of miR-429/miR-141-3p post-NAC; and (4) a distinct pre-NAC miRNA profile in the patient with the lowest residual disease (Luminal B, RCB-I).

The absence of statistically significant results after FDR correction is unsurprising and should be interpreted as a power issue rather than a negative result. With *n* = seven pairs, the two-sided Wilcoxon signed-rank test cannot return an uncorrected *p*-value below 1/2^6^ = 0.0156, which is the exact minimum attainable *p*-value for this design; after Benjamini–Hochberg correction across 759 tests, this floor corresponds to an adjusted *p*-value of approximately 0.46, so no result could have reached *p*adj < 0.05 irrespective of effect size. This is a structural property of the test at this sample size rather than an empirical finding, and it is the reason why exploratory thresholds were pre-specified. The use of relaxed thresholds combined with an effect-size filter for candidate prioritisation in underpowered discovery studies, followed by independent confirmation, is consistent with the recommendations of Button et al. [21] and with the REMARK reporting framework [22].

### 3.2. Biological Plausibility of the Leading Candidates

The post-NAC upregulation of hsa-miR-139-3p and hsa-miR-139-5p is particularly noteworthy biologically. miR-139-5p has been repeatedly characterised as a tumour suppressor. In breast cancer cells, Sun et al. showed that miR-139-5p directly targets the 3′-UTR of NOTCH1, that its enforced expression reduces viability, migration and invasion, and that it modulates sensitivity to docetaxel [14]. Direct repression of NOTCH1 by miR-139-5p has been independently confirmed in colorectal carcinoma, where it was accompanied by inhibition of MMP-7 and MMP-9 and by induction of apoptosis and G0/G1 arrest [15]; in hepatocellular carcinoma, miR-139 suppresses metastasis through downregulation of Rho-kinase 2 [16]. In breast cancer specifically, miR-139-5p has been described as a regulator of metastatic pathways [17]. Our observation of a consistent post-NAC increase in both miR-139-5p and miR-139-3p across all seven pairs is therefore directionally concordant with a chemotherapy-associated shift towards a less aggressive residual phenotype, although the present design cannot distinguish induction within surviving tumour cells from selection, or from a change in the cellular composition of the residual tumour bed.

The loss of miR-200-family members after NAC is likewise consistent with an established mechanism. miR-429 and miR-141-3p belong to the family whose members directly repress the E-cadherin transcriptional repressors ZEB1 and ZEB2 [18,19], with which they form a double-negative feedback loop [20]; their loss therefore permits E-cadherin downregulation and acquisition of a mesenchymal phenotype. Selection for such a phenotype under chemotherapy would be expected to associate with residual disease, and six of our seven patients had RCB-II or RCB-III disease [23,24].

One aspect of our data does not fit a simple interpretation in which chemotherapy uniformly activates tumour suppressor programmes, and we report it explicitly. miR-708-5p and miR-708-3p, and let-7a-3p, were downregulated post-NAC, yet miR-708 has been characterised as a tumour suppressor in breast cancer and the let-7 family is among the most consistently reported tumour-suppressive miRNA families. The candidate signature is therefore directionally mixed, and any interpretation framed solely as chemotherapy-induced activation of antitumour programmes would be selective. Distinguishing genuine transcriptional modulation in surviving tumour cells from selection of pre-existing clones, and from the substantial change in stromal and immune cellular composition of the post-NAC tumour bed, will require single-cell or spatially resolved profiling rather than bulk sequencing of residual tissue.

The distinct pre-NAC miRNA profile observed in patient P3 (Luminal B, RCB-I)—the only patient achieving a near-complete response—is conceptually consistent with the hypothesis that pre-treatment miRNA expression may discriminate between chemosensitive and chemoresistant tumours. However, with *n* = 1 in the “best responder” category, this observation is purely hypothesis-generating and requires validation in larger cohorts stratified by RCB class.

### 3.3. Comparison with Published miRNA Studies in Breast Cancer

Our findings can be placed alongside three bodies of published work. Xing et al. [11] derived a miRNA signature predictive of neoadjuvant chemotherapy response in breast cancer; their design differs from ours in that it compared responders with non-responders at baseline, whereas we compared each tumour with itself before and after treatment. The two designs answer complementary questions—theirs identifies a predictive signature, ours a pharmacodynamic one—and the observation that our single best responder was also the patient with the most distinct baseline profile is consistent with, but does not replicate, their finding. Apollonova et al. [12] reported that resistance of breast cancer cells to paclitaxel is associated with low expression of miR-186 and miR-7; neither miRNA met our exploratory thresholds, which may reflect the difference between a cell-line model of acquired resistance and residual disease in patients treated with combination regimens, or simply the limited power of our analysis.

The most directly comparable clinical dataset is that of Lindholm et al. [25], who profiled miRNA expression in 241 biopsies from 132 patients sampled before treatment, after anthracycline and after taxane in a randomised neoadjuvant trial. They reported that tumour suppressor miRNAs were upregulated after treatment while oncogenic miRNAs were downregulated, and that deregulated miRNA expression correlated with a decrease in proliferation score during treatment. The direction of our leading finding—post-NAC upregulation of the tumour suppressor miR-139 family—is concordant with that pattern in a cohort two orders of magnitude smaller, which we regard as the most encouraging aspect of the present dataset.

Placing an exploratory list of this kind against the aggregate evidence is sobering and, we believe, necessary. In a systematic review and meta-analysis of 59 studies, Zhang et al. found that 60 of 123 miRNAs examined had been reported as related to NAC response in breast cancer, yet only a small minority were reproducible enough to enter a quantitative synthesis; elevated baseline tissue miR-7 was the single marker associated with a higher pCR rate across studies [26]. Two conclusions follow for the present work. The first is that the field’s problem is not a shortage of candidate miRNAs but a shortage of candidates that survive independent replication, which is precisely why we present ours as a prioritised list rather than as a signature. The second is that miR-7, the most reproducible candidate in that synthesis, did not meet our exploratory thresholds; with *n* = 7, this is uninformative about miR-7 itself, but it is a useful reminder that the absence of an established marker from a list of this size carries no evidential weight in either direction.

The reliability of miRNA quantification in archival FFPE material has been examined directly and is relevant to the interpretation of our workflow. In matched frozen/FFPE pairs from nine tumours profiled by ligation-based deep sequencing, Meng et al. reported per-sample correlations of miRNA expression values between the two preservation methods of r = 0.71–0.95, with storage times of two to nine years not affecting the number of miRNAs detected [27]. Kakimoto et al. similarly found that global miRNA expression profiles in matched frozen and FFPE tissue were highly correlated (0.88 < r < 0.92), but demonstrated that degradation is not uniform across miRNAs: species with GC content below 40% are preferentially lost, which distorts abundance rankings within a library [28]. Two consequences follow. First, comparisons of relative abundance between different miRNAs in our dataset should be treated with caution. Second, and more favourably for the present design, both specimens from a given patient underwent the same fixation and embedding workflow, so a GC-dependent degradation bias affects the pre- and post-NAC libraries of a pair in the same direction and is largely cancelled in the within-pair difference. A recent systematic review and feasibility study focused specifically on archival FFPE breast cancer tissue reached a similar conclusion for genomic analyses more broadly, while emphasising that block age, fixation duration and cold ischaemia time are the pre-analytical variables that most consistently degrade data quality and that they should be recorded and reported rather than assumed to be uniform [29].

### 3.4. Clinical Relevance

Although exploratory, paired longitudinal profiling offers information that cannot be obtained from cross-sectional studies. Monitoring dynamic miRNA changes induced by neoadjuvant chemotherapy may identify biomarkers reflecting treatment adaptation rather than baseline tumour biology alone. Such longitudinal biomarkers could eventually complement established pathological endpoints such as RCB and improve response monitoring, particularly in the substantial group of patients who do not achieve a pathological complete response and for whom post-neoadjuvant treatment escalation is currently guided by residual disease burden alone.

Two further considerations follow from the paired design itself. First, the value of paired sampling is that each tumour serves as its own control: inter-patient variability in baseline miRNA expression, which dominates the global variance in our own PCA and is the principal source of noise in cross-sectional biomarker studies, is removed by construction. This is what allows a seven-patient study to detect perfectly consistent directional change at all, and it is the main methodological argument for collecting post-treatment specimens systematically rather than opportunistically. Second, tissue-based and circulating approaches are complementary rather than competing. Meta-analytic work on circulating miRNAs in the neoadjuvant setting has identified miR-21-5p and miR-34a-5p as the most consistently reported non-invasive candidates, while also documenting substantial between-study heterogeneity in pre-analytical handling, normalisation strategy and reporting that currently prevents clinical implementation [30,31]. A tissue-anchored pharmacodynamic signature of the kind explored here could in principle be used to select which circulating species are worth monitoring serially, which is a more tractable path than screening the circulating compartment de novo.

### 3.5. Limitations

Several limitations of this study must be acknowledged. First, the small sample size (*n* = 7) is the dominant limitation, precluding definitive statistical conclusions and meaningful subgroup analyses by molecular subtype. Second, the absence of pCR in any patient reduces the discriminatory power of the analysis; future studies should target cohorts enriched for both pCR cases and RCB-III cases. Third, the methodological heterogeneity of NAC regimens (AC-T vs. TCHP) introduces a confounding factor that cannot be controlled in a seven-patient cohort.

Fourth, because of the limited sample size, subtype-specific analyses were not statistically feasible, and all analyses should therefore be interpreted as exploratory. Pooling four molecular subtypes in a seven-patient paired analysis necessarily forfeits subtype-specific resolution: the consistency of direction observed for the leading candidates across all seven pairs indicates that the changes are not driven by a single subtype, but it does not establish that the magnitude of change is subtype-independent. Future validation should be performed separately within homogeneous molecular subtype cohorts.

Fifth, no candidate was confirmed by an orthogonal method. A candidate list derived from 759 parallel tests in seven pairs is expected to contain false positives, and the uniform *p* = 0.0156 shared by the top-ranked miRNAs in Table S1 is the exact minimum attainable value of the Wilcoxon signed-rank test at *n* = 7 rather than a measure of the strength of evidence: it is obtained by any miRNA that changes in the same direction in all seven pairs, whatever the magnitude of that change. Under the null hypothesis, approximately 12 of 759 miRNAs would be expected to show perfectly consistent directionality by chance alone; the log_2_ fold change filter reduces but does not eliminate this expectation. Due to the limited amount of remaining FFPE-derived RNA after library preparation, RT-qPCR validation could not be performed in the current pilot study. Validation of the candidate miRNAs by RT-qPCR, with MIQE-compliant reporting [32], is planned as the first step of the ongoing expanded cohort [33], and none of the 156 candidates reported here should be regarded as a validated biomarker in the interim.

Sixth, although FFPE tissues represent routine clinical practice and are well suited for miRNA profiling because of the intrinsic stability of miRNAs [27,28], future studies should validate these findings in prospectively collected fresh-frozen specimens. We note in addition a systematic feature of the paired design that is not resolved by the within-pair comparison: the pre-NAC specimen is a core needle biopsy fixed almost immediately, whereas the post-NAC specimen is a surgical resection with a longer and more variable cold ischaemia and fixation time. This difference is present in every pair and in the same direction, and it is a genuine confounder of the pre-to-post comparison that cannot be resolved in the present dataset; prospective collection with standardised cold ischaemia times is therefore a prerequisite for the confirmatory study.

Despite these limitations, this study offers several contributions: it validates the technical feasibility of paired small-RNA sequencing from clinical FFPE specimens in a Romanian oncology setting; it establishes the analytical pipeline (miRge3.0, paired Wilcoxon, BH correction, Spearman correlations) that will be applied to the expanded cohort (*n* = 149, Komporaly et al., Cureus 2026) [33]; and it generates biologically plausible candidate miRNAs for RT-qPCR validation [32]. Integrating miRNA profiling with RCB quantification represents a methodological advance over studies that use binary pCR as the sole endpoint, because RCB captures the full spectrum of treatment response.

## 4. Materials and Methods

### 4.1. Study Design and Patient Selection

This retrospective–translational pilot study was conducted at Memorial Băneasa Hospital, Bucharest, Romania, with molecular analysis performed at CeGaT GmbH (Research & Pharma Solutions, Tübingen, Germany). The study was conducted in accordance with the Declaration of Helsinki.

Ethical approval was obtained from two committees covering the two components of the study: the Ethics Committee of the “Prof. Dr. Alexandru Trestioreanu” Institute of Oncology, Bucharest (approval no. 1/19.01.2023), covering the clinical data component, and the Ethics Committee of Memorial Hospital, Bucharest (approval no. 471/13.10.2025), covering the FFPE specimen molecular sub-study. All patient data were anonymised before analysis.

Eligibility criteria included: (1) histologically confirmed invasive breast carcinoma; (2) completion of full NAC followed by definitive surgery; (3) availability of a paired pre-NAC core needle biopsy and post-NAC surgical specimen in FFPE format; and (4) availability of complete clinicopathological data, including RCB assessment. All molecular subtypes were eligible provided NAC had been administered. Patients with Luminal A tumours were included when NAC had been indicated on the basis of nodal involvement or tumour size precluding upfront breast-conserving surgery, which accounts for the three Luminal A cases in the final cohort. Seven patients meeting all criteria were identified from the institutional database (2021–2025).

### 4.2. Clinical and Pathological Assessment

Molecular subtype was determined by immunohistochemistry (IHC) on diagnostic biopsy specimens: oestrogen receptor (ER), progesterone receptor (PR), HER2, and Ki-67, according to the St. Gallen 2021 consensus recommendations [13]. Cases with IHC HER2 2+ were confirmed by fluorescence in situ hybridisation (FISH). NAC regimens were assigned according to institutional protocols: anthracycline–taxane-based (AC-T, 8 cycles) for Luminal and TNBC disease, and TCHP (docetaxel, carboplatin, trastuzumab, pertuzumab, 6 cycles) for HER2-positive disease. All TNBC patients received pembrolizumab according to the KEYNOTE-522 protocol.

Pathological complete response was defined as ypT0/is ypN0 (absence of residual invasive carcinoma in the breast and axillary nodes). The Residual Cancer Burden (RCB) score and class (RCB-0/I/II/III) were calculated using the validated MD Anderson online calculator (www.mdanderson.org/breastcancer_RCB, accessed on 10 June 2026) based on four parameters: residual tumour-bed dimensions, percentage cellularity of the invasive carcinoma, percentage of in situ carcinoma, and the number and size of nodal metastases.

### 4.3. RNA Extraction and Small-RNA Sequencing

Total RNA was extracted from FFPE sections (10 µm thickness, 2–4 sections per specimen) using the MagMAX™ FFPE DNA/RNA Ultra Kit (Thermo Fisher Scientific, Waltham, MA, USA) according to the manufacturer’s protocol. RNA quality was assessed by fluorometric quantification (Qubit RNA HS Assay) and fragment-length analysis (DV200 parameter, Agilent TapeStation, Santa Clara, CA, USA). The minimum DV200 threshold for inclusion was 20%, consistent with CeGaT quality standards for FFPE-derived RNA.

Small-RNA sequencing libraries were prepared using the QIAseq miRNA Library Kit (Qiagen, Venlo, The Netherlands), incorporating unique molecular identifiers (UMIs) for PCR-duplicate removal. Libraries were sequenced on the NovaSeq X Plus platform (Illumina, San Diego, CA, USA) at CeGaT GmbH, Tübingen, Germania (project S17293, 3–8 May 2026) at approximately 10 million reads per sample (single-end, 50 bp). The mean Q30 sequencing-quality value across all samples was 92.76%.

### 4.4. Bioinformatic Analysis

Raw FASTQ files were demultiplexed using Illumina bcl2fastq (v2.20) and quality-assessed using FastQC on the DRAGEN Bio-IT platform (v4.4.4). Adapter trimming and miRNA alignment were performed using miRge3.0 (Patil & Halushka, 2021) [34] against the human reference genome (GRCh38) and miRNA annotations from miRBase v22 (Kozomara & Griffiths-Jones, 2013) [35]. Raw miRNA counts were extracted from the miR.Counts.tsv output files.

For differential expression analysis, miRNAs not meeting the filtering threshold: ≥3 counts in ≥3 of 14 samples, yielding 759 analysable miRNAs. Differential expression between paired pre- and post-NAC samples was assessed using the Wilcoxon signed-rank test (two-sided, paired design, *n* = 7 pairs), implemented in Python 3.9 (scipy.stats). Multiple testing correction was applied using the Benjamini–Hochberg (BH) false discovery rate (FDR) procedure. Both strict significance criteria (*p*adj < 0.05, |log_2_FC| > 1.0) and exploratory criteria (uncorrected *p* < 0.10, |log_2_FC| > 0.5) were pre-specified, the latter being standard practice for pilot biomarker studies.

Because the primary analysis is a paired within-patient comparison, constitutive between-patient and between-subtype differences in baseline miRNA expression are removed by the design; the quantity tested is the consistency of the direction and magnitude of the pre-to-post change across the seven pairs. The analysis does not, and cannot, address whether the magnitude of that change differs between molecular subtypes, which would require a subtype-stratified design and a substantially larger cohort.

Principal component analysis (PCA) was performed on variance-stabilised log_2_(RPM+1)-transformed expression values (sklearn.decomposition.PCA, StandardScaler normalisation). Spearman rank correlation coefficients (ρ) and corresponding *p*-values were calculated to assess associations between miRNA expression levels and continuous clinical variables (RCB score, Ki-67, tumour size, clinical timing intervals). All statistical analyses were performed using Python 3.11 (scipy, pandas, sklearn, seaborn, matplotlib). All *p*-values are two-sided; significance thresholds are reported with the corresponding interpretation.

### 4.5. Functional Annotation of Candidate miRNAs

Candidate miRNAs meeting the exploratory criteria were annotated functionally using experimentally validated miRNA–target interactions retrieved from miRTarBase (release 9.0) and confirmed against the primary literature; only interactions supported by strong experimental evidence (luciferase reporter assay, Western blot, or qRT-PCR of the target transcript) were retained, and predicted-only interactions were excluded. Because the candidate list was derived under exploratory thresholds and did not survive multiple testing correction, no formal over-representation or pathway enrichment statistics were computed; the resulting network (Figure 8) is presented as a literature-curated functional summary of the observed signature and not as the output of a statistical enrichment analysis.

## 5. Conclusions

This pilot translational study demonstrates the technical feasibility of paired pre/post-NAC profiling by small-RNA sequencing on FFPE specimens in a real-world clinical setting and identifies 156 candidate miRNAs modulated by neoadjuvant chemotherapy in breast cancer. The leading candidates—including miR-139-3p/5p (upregulated) and miR-429/miR-141-3p (downregulated) post-NAC—are biologically plausible and concordant with the existing literature on miRNA-mediated chemoresistance mechanisms.

These candidates are, however, derived from a single platform in seven patients; none reached statistical significance after multiple testing correction, and none have been confirmed by an orthogonal method or in an independent cohort; they should therefore be regarded as a prioritised list for confirmation rather than as results in their own right. Their appropriate use is as the pre-specified target set for an adequately powered prospective study with subtype-stratified analysis, RT-qPCR confirmation and prospectively collected specimens, which represents the direct continuation of the present work.

## Figures and Tables

**Table 1:** Clinicopathological characteristics, treatment parameters, and outcomes of the seven patients.

No.	Age/Subtype	cTN	Regimen	ypTN	RCB Class (Score)	Days Diag → NAC	Days NAC → Surgery
P1	TNBC, G3, Ki-67 85%	T2N0	AC-T (8/8)	ypT2 ypN1	III (3.700)	21	27
P2	Luminal A, G2, Ki-67 10%	T1cN1	AC-T (8/8)	ypT1b ypN1a	III (3.630)	26	35
P3	Luminal B, G2, Ki-67 45%	T2N1	AC-T (8/8)	ypT1a ypN0	I (1.320)	11	36
P4	Luminal A, G2, Ki-67 15%	T2N1	AC-T (7/8) *	ypT1c ypN1a	III (3.660)	67	33
P5	TNBC, G3, Ki-67 20%	T3N1	AC-T (6/8) **	ypT2 ypN1a	II (3.210)	21	90
P6	Luminal A, G2, Ki-67 5%	T1N1	AC-T (8/8)	ypT1b ypN0	II (1.600)	23	34
P7	HER2+, G2, Ki-67 30%	cT2N1	TCHP (6/6)	ypT2 ypN1	III (3.506)	20	15

* Dose reduction (80%) due to an allergic reaction. ** 6/8 cycles completed due to grade ≥ 3 neutropenia (dose intensity 90%). Luminal B defined as HR+/HER2− with Ki-67 ≥ 20% or low PR [13]. RCB: Residual Cancer Burden class and score (MD Anderson calculator). All patients: ECOG 0; all tumours: invasive carcinoma NST; all: R0 resection margins.

## Data Availability

The data presented in this study are available on reasonable request from the corresponding author. The data are not publicly available owing to patient-privacy restrictions.

## Supplementary Materials

The following supporting information can be downloaded at: https://www.mdpi.com/article/10.3390/ijms27177799/s1.
