# Supplementary material for: MicroRNA Expression Profiles Before and After Neoadjuvant Chemotherapy in Breast Cancer: Correlations with Molecular Subtypes, Pathological Response, and Clinical Timing—A Pilot Translational Study
Source: Int J Mol Sci. 2026 Aug 31;27(17):7799. doi: 10.3390/ijms27177799 (PMC13566632; doi:10.3390/ijms27177799)
Supplement: Supplementary file 1 [file ijms-27-07799-s001.zip › ijms-4448287-supplementary.pdf]

International Journal of *Molecular Sciences*

MDPI

Article

MicroRNA Expression Profiles Before and After Neoadjuvant Chemotherapy in Breast Cancer: Correlations with Molecular Subtypes, Pathological Response, and Clinical Timing—A Pilot Translational Study

Isabela Anda Komporaly [1,2], Adelina Silvana Gheorghe [3,*], Elena Adriana Iovănescu [1], Bogdan Georgescu [1,4] and Dana Lucia Stănculeanu [1,3]

[1] Department of Oncology, "Carol Davila" University of Medicine and Pharmacy, 020021 Bucharest, Romania; isabelakomporaly@gmail.com (I.A.K.); dumitrescu.elena.adriana@gmail.com (E.A.I.); dr_bogdan.georgescu@icloud.com (B.G.); dlstanculeanu@gmail.com (D.L.S.)
[2] Memorial Băneasa Hospital, 013812 Bucharest, Romania
[3] Department of Medical Oncology I, "Prof. Dr. Alexandru Trestioreanu" Institute of Oncology, 022328 Bucharest, Romania
[4] Neolife Hospital, 077190 Bucharest, Romania
* Correspondence: adelina.silvana.gheorghe@gmail.com

Table S1. Top 20 differentially expressed miRNAs (ranked by Wilcoxon p-value, paired pre- vs. post-NAC, n = 7).

Rank	miRNA	log_2FC (Post−Pre)	p-value (Wilcoxon)	padj (BH)	Direction
1	hsa-let-7a-3p	−1.023	0.0156	0.456	↓ post-NAC
2	hsa-miR-874-5p	+1.182	0.0156	0.456	↑ post-NAC
3	hsa-miR-1277-5p	−1.254	0.0156	0.456	↓ post-NAC
4	hsa-miR-874-3p	+1.229	0.0156	0.456	↑ post-NAC
5	hsa-miR-708-3p	−0.791	0.0156	0.456	↓ post-NAC
6	hsa-miR-708-5p	−1.177	0.0156	0.456	↓ post-NAC
7	hsa-miR-1323	+1.551	0.0156	0.456	↑ post-NAC
8	hsa-miR-130a-3p	+0.932	0.0156	0.456	↑ post-NAC
9	hsa-miR-139-3p	+2.603	0.0156	0.456	↑ post-NAC
10	hsa-miR-139-5p	+2.357	0.0156	0.456	↑ post-NAC
11	hsa-miR-141-3p	−1.795	0.0156	0.456	↓ post-NAC
12	hsa-miR-140-3p	+1.072	0.0156	0.456	↑ post-NAC
13	hsa-miR-429	−2.526	0.0156	0.456	↓ post-NAC
14	hsa-miR-665-3p	+1.485	0.0156	0.456	↑ post-NAC
15	hsa-miR-374b-5p	−0.477	0.0156	0.456	↓ post-NAC
16	hsa-miR-3609	+0.624	0.0156	0.456	↑ post-NAC
17	hsa-miR-4458	−0.531	0.0156	0.456	↓ post-NAC
18	hsa-miR-454-3p	+0.718	0.0156	0.456	↑ post-NAC
19	hsa-miR-548av-5p	+0.852	0.0156	0.456	↑ post-NAC
20	hsa-miR-196a-5p	−0.694	0.0156	0.456	↓ post-NAC

padj: Benjamini–Hochberg-adjusted p-value. Note: the uniform p-value (p = 0.0156 = 1/64) reflects the minimum achievable p-value of the Wilcoxon signed-rank test for n = 7 pairs; several miRNAs share this minimum value because all showed consistent directionality across all 7 pairs. FC: fold change. All values are exploratory; formal significance requires padj < 0.05, which was not reached in this n = 7 pilot.
